# Cardiovascular magnetic resonance predictors of clinical outcome in patients with suspected acute myocarditis

**DOI:** 10.1186/s12968-015-0185-2

**Published:** 2015-08-29

**Authors:** Francesca Sanguineti, Philippe Garot, Melina Mana, Darach O’h-Ici, Thomas Hovasse, Thierry Unterseeh, Yves Louvard, Xavier Troussier, Marie-Claude Morice, Jérôme Garot

**Affiliations:** The Hôpital Privé Jacques Cartier – ICPS, CMR Department, Ramsay-Générale de Santé, 6 Avenue du Noyer Lambert, 91300 Massy, France

**Keywords:** Cardiovascular magnetic resonance, Myocarditis, Outcome

## Abstract

**Background:**

The natural history of acute myocarditis (AM) remains highly variable and predictors of outcome are largely unknown. The objectives were to determine the potential value of various cardiovascular magnetic resonance (CMR) parameters for the prediction of adverse long-term outcome in patients presenting with suspected AM.

**Methods:**

In a single-centre longitudinal prospective study, 203 routine consecutive patients with an initial CMR-based diagnosis of AM (typical Late Gadolinium Enhancement, LGE) were followed over a mean period of 18.9 ± 8.2 months. Various CMR parameters were evaluated as potential predictors of outcome. The primary endpoint was defined as the occurrence of at least one of the combined Major Adverse Clinical Events (MACE) (cardiac death or aborted sudden cardiac death, cardiac transplantation, sustained documented ventricular tachycardia, heart failure, recurrence of acute myocarditis, and the need for hospitalization for cardiac causes).

**Results:**

The vast majority of patients (*N* = 143,70 %) presented with chest pain, mild to moderate troponin elevation and ST-segment or T wave abnormalities. Various CMR parameters were evaluated on initial CMR performed 3 ± 2 days after acute clinical presentation (LV functional parameters, presence/extent of edema on T2 CMR, and extent of late gadolinium enhancement lesions). Out of the 203 patients, 22 experienced at least one major cardiovascular event (10.8 %) during follow-up for a total of 31 major cardiovascular events. Among all CMR parameters, the only independent CMR predictor of adverse clinical outcome by multivariate analysis was an initial alteration of LVEF (*p* = 0.04).

**Conclusions:**

In routine consecutive patients without severe hemodynamic compromise and a CMR-based diagnosis of AM, various CMR parameters such as the presence and extent of myocardial edema and the extent of late gadolinium-enhanced LV myocardial lesions were not predictive of outcome. The only independent CMR predictor of adverse clinical outcome was an initial alteration of LVEF.

## Background

Acute myocarditis (AM), which is most frequently caused by a viral infection [1, 2], is characterized by heterogeneous clinical manifestation, including chest pain, heart failure, arrhythmias, or a combination of these. Although it has been recognized in pathologic studies to be associated with 8.6 to 12 % of sudden cardiac deaths [3, 4], and up to 9 % of dilated cardiomyopathy [1, 5, 6], its clinical presentation is less severe in the vast majority of the cases and heart failure is uncommon. [7–9] Because the natural history of AM remains highly variable, the prognostic stratification is of critical importance.

Endomyocardial biopsy (EMB) remains the gold standard for the diagnosis of AM but is recommended for the most severe patients, namely those with acute dilated cardiomyopathy and hemodynamic compromise, life-threatening arrhythmias, or those requiring circulatory support, thus excluding the vast majority of patients presenting with AM in routine practice [10]. EMB is an invasive procedure that may be hampered by a relatively low sensitivity, because of the focal nature of the disease [6, 11]. Also, it is limited by interobserver variability and lacks prognostic value [6, 12]. Cardiovascular Magnetic Resonance (CMR) has emerged as the most important non-invasive imaging technique for the diagnosis of AM [13]. Besides the assessment of functional parameters such as left ventricular (LV) function and volumes, it provides comprehensive analysis of myocardial inflammatory process and accurate depiction of various patterns of irreversible myocardial damage with unprecedented diagnostic value [13, 14]. At the acute stage of AM, several predictors of outcome have been postulated such as ventricular dysfunction [7, 9], advanced New York Heart Association class [15, 16], elevated pulmonary artery pressure [16], prolonged QRS duration [17], specific histopathological forms or patterns of tissue damage [18, 19], increased myocardial early gadolinium enhancement ratio [20], or presence of late gadolinium enhancement [21]. However, these predictors are still controversial [1, 9], and in particular those extracted from CMR. The aim of this study was to investigate the potential value of various CMR parameters for the prediction of adverse long-term outcome in patients presenting with suspected AM in routine practice.

## Methods

### Screened patients

Between October 2008 and December 2011, we conducted a single-centre longitudinal prospective non-randomized study in consecutive patients presenting with a first episode of suspected AM at our Institutions (CMR Laboratory in a tertiary Cardiovascular Centre). To participate at the screening phase, patients were eligible if they had a clinical presentation suggesting the diagnosis of AM. Patients who had hat least 3 of the following criteria: 1) chest pain, 2) recent history (<1 month) of acute viral infection, 3) abnormal ST segment or T waves on ECG, 4) troponin elevation, were eligible to undergo CMR at the time of acute clinical presentation. Patients presenting with ST segment elevation >1 mm in at least 2 contiguous derivations were scheduled for prompt coronary angiography. They were eligible for the CMR study if the coronary angiogram did not show any significant (>50 % diameter stenosis) coronary disease. Patients were then eligible to participate in the study if initial CMR showed nonischemic suggestive patterns of AM as defined below and if the patient had no contraindication to CMR.

Exclusion criteria were: 1) severe noncardiac disease compromising life expectancy during the period of the study 2) pre-existing other cardiac disease, 3) relevant coronary artery disease (coronary stenosis ≥50 %) proven by angiography, 4) severe hemodynamic compromise that precluded the CMR study. All patients gave informed written consent and the study was approved by the Local Ethic Committee of our Institutions.

### Study protocol

The patients eligible for participation in the study were scheduled to undergo CMR at our Institutions between day 0 and day 7 after initial presentation with a detailed CMR protocol as described below. When the diagnosis of AM was made on CMR, medical treatment included ASA (3 g per day), β-blockers and ACE inhibitors for at least 6 weeks. Supporting therapy was provided when appropriate in case of acute or subacute heart failure. Patients with diagnosis of AM were then included after informed consent and scheduled for a follow up assessment between 12 and 18 months after the initial event. The follow up consisted of a clinical visit for patients who were initially hospitalized in our Institutions or a direct contact with the patient and the referring cardiologist for patients who were initially referred to our CMR suite from other hospitals. For all patients, a clinical questionnaire with a detailed description of clinical study endpoints was thoroughly filled out by two senior cardiologists (FS, JG) in charge of the follow up assessment. The LV function was assessed by CMR when available or by echocardiography between 6 months and one year after initial presentation.

### Study end-points

Patients were followed over time and the primary clinical end-point was the occurrence of at least one of the combined Major Adverse Clinical Events (MACE) defined by: cardiac death (or aborted sudden cardiac death), cardiac transplantation, sustained documented ventricular tachycardia (documented by Holter ECG or ECG in symptomatic patients), the need for hospitalization for heart failure (NYHA class IV and III), recurrence of acute myocarditis, and the need for hospitalization for cardiac causes. Clinical event adjudication was based on the follow up clinical visit or contact, with a consensus reached by the 2 senior cardiologists in charge of follow up (FS, JG). The secondary end-points were the LV ejection fraction (EF) on last echocardiography (by Simpson’s biplane analysis) or on CMR (the intra-individual comparisons were made by the same method), and the persistence of clinical symptoms as defined by a clinical score as follows: 1 point for chest pain, palpitations, arrhythmia, asthenia; 1 point for each NYHA functional class; 1 point for the need of a referral to the cardiologist; and 3 points for hospitalization.

### CMR acquisitions

In eligible patients, CMR was promptly performed between day 0 and day 7 after acute clinical presentation in a dedicated Cardiovascular MR laboratory on a Siemens Magnetom Espree® 1,5 T scanner (Erlangen, Germany) with 32-channel anterior and spinal coils for reception. Long-axis (2-, 3-, and 4-chamber) and short-axis cine MR images encompassing the left ventricle from base to apex were obtained with a retrospectively gated fast imaging Steady State Free Precession (SSFP) sequence. Myocardial edema and inflammation was investigated in matched long axis 2-, 3-, and 4—chamber, and in short-axis views through T2-weigthed triple inversion-recovery turbo spins echo sequence with inversion pulses for fat and blood suppression. Then, a bolus of gadolinium contrast (Dotarem®, Guerbet, Aulnay, France, 0.1 mmol/kg) was injected at a rate of 4 ml/s with an injector (Optistar Elite Mallinckrodt). Focal early gadolinium enhancement (EGE) was visually assessed on SSFP cine-MR images in the long axis 2-, 3-, 4-chamber views and short-axis locations, acquired between 2 and 5 min. after Gadolinium injection. Late gadolinium enhanced (LGE)-CMR was acquired 10 min. after contrast administration in matched long axis 2-, 3-, 4- chamber and short-axis views through the use of spoiled 2D fast gradient echo inversion-recovery sequence with a TI set to null normal myocardial signal (TI scout sequence). The diagnosis of AM was made based on the following criteria: 1) spontaneous intramyocardial and/or supepicardial hypersignal on T2-weighted spin echo images indicative of myocardial edema, 2) intramyocardial and/or supepicardial LGE on CMR images, 3), absence of microvascular obstruction and acute myocardial infarction (MI) on CMR images. The criteria 2 and 3 were mandatory for the diagnosis of AM and participation to the study.

When the diagnosis of AM was made, medical treatment included ASA (3 g per day), β-blockers and ACE inhibitors for at least 6 weeks. Supporting therapy was provided when appropriate in case of acute or subacute heart failure.

### CMR analysis

CMR images were analyzed by two blinded experienced observers and in case of discordance by a third observer for consensus. Myocardial segmentation was assessed using the 17-segment model according to the consensus of the North American Society of Myocardial Imaging. Endocardial and epicardial borders were manually traced on end-diastolic and end-systolic short-axis cine images from base to apex. LV volumes and ejection fraction were derived by summation of epicardial and endocardial contours (Argus® Software, Siemens, Erlangen, Germany). Myocardial edema was assessed as a hypersignal that was present before contrast injection on black-blood T2-weighted images. The extent of myocardial edema on T2-weighted images, of early gadolinium enhancement (EGE) on cine images and of late gadolinium enhancement (LGE) was assessed through the use of a semi-quantitative analysis. Each of the 17 LV myocardial segments was divided into 3 layers (outer, mid and inner). The presence of myocardial lesions was determined in each of the 3 layers for each myocardial segment. Finally, myocardial lesions on CMR data were delineated by planimetry using an automated threshold greater than 4SD above mean myocardial signal intensity and expressed as percentage of LV myocardial surface area. Myocardial damage on LGE-CMR was localized in details in the sub-epicardium, midwall or transmural and its shape was characterized as nodular or linear.

### Statistical analysis

Statistical analysis was performed using STATA 10.1 (Statacorp LP, Texas USA). Baseline descriptive statistics are presented as frequency and percentage for categorical variables and mean ± SD or median (interquartile range) for continuous variables. The normality of data was assessed using the Skewness and Kurtosis normality test. The differences between the groups were assessed with the chi-square test or Fisher exact test for categorical data and Student *t*-test for continuous data. Differences between groups were considered statistically significant if the 95 % CI around the difference of the means did not contain zero. In a prespecified analysis, baseline predictors for MACE were identified using Cox multivariate analysis including variables showing a *p* value <0.20 in association with MACE by univariate analysis. In the multivariate analysis, colinearity was considered if Pearson’s correlation coefficient was > 0.6 or the standard error of a covariate was > 5.0. If colinearity was identified, the multivariate analysis was repeated after removal of the responsible covariate.

## Results

### Study population

Overall, 240 patients were screened to participate. Six of them had contra-indications to MRI (claustrophobia in 4, metallic ocular implants in 1, cerebral aneurysm clip in 1) and only one refused to participate. Thus, 233 patients were considered for the study. Out of them, 28 patients were excluded because of the presence of relevant coronary artery disease in 8, severe hemodynamic compromise in 3 (cardiogenic shock), myocardial involvement in inflammatory systemic diseases in 5 (including sarcoidosis, Behcet, Churg Strauss, Lyme disease or sepsis), other concomitant cardiac diseases in 9 (chemotherapy-induced cardiomyopathy, valve disease, arrhythmogenic right ventricular dysplasia), and pericardial diseases in 3. Finally, 205 patients were enrolled in the study and followed over time. Two patients were lost to follow-up and the final study population consisted of 203 patients. The baseline characteristics of patients are detailed in Table 1. The vast majority of patients (*N* = 143, 70 %) presented with a clinical scenario including chest pain, mild to moderate troponin elevation and ST-segment or T wave abnormalities on ECG. Out of these 143 acute coronary syndrome-like patients, 99 (69 %) had prompt normal coronary angiography because of ST segment elevation in at least 2 contiguous derivations. In the 44 remaining patients (31 %), coronary angiography was not performed because of the lack of ST segment elevation and a very low risk profile (mean age 40 years, no cardiovascular risk factors). The remaining 60 patients had a presentation that included for the majority of them a clear recent history of acute viral infection with fever (38/60), and the characteristics of chest pain were highly suggestive of non ischemic origin (variable with respiratory motion, relieved by aspirin). Ten patients presented with mild or moderate heart failure, 12 with arrhythmias. All patients were relatively young and had no cardiovascular risk factors. They all had typical patterns of acute non ischemic lesions on LGE-CMR (multiple, nodular, subepicardial) and for those reasons were not referred to cath. The other symptoms at initial presentation are listed in Table 1. The clinical score at presentation was 4.6 ± 1.5.

**Table 1:** Baseline characteristics of study patients

	N (203)
Age, yrs	42.7 ± 16.5
Male (N,%)	155 (76)
History of cardiovascular disease (N,%)	4 (2)
Time between symptoms onset and CMR (days)	3 ± 2
Time between CMR and subsequent assessment of LV EF (months)	9.8 ± 6.8
Time between CMR and follow-up (months)	18.9 ± 8.2
Initial clinical score	4.6 ± 1.5
Clinical presentation at acute stage (N,%)	
Chest pain - ST and/or T ECG changes - elevated troponin	143 (70)
Chest pain – recent history of viral infection – elevated troponin	22(11)
Chest pain – recent history of viral infection – ST and/or T ECG changes	16(8)
Sustained ventricular arrhythmias, cardiac arrest	12 (6)
Heart failure	10 (5)

Data are expressed as mean ± SD unless specified

### CMR data

The initial CMR findings are listed in Table 2. CMR was performed in all patients at a mean of 3 ± 2 days after acute clinical presentation. Mean LVEF was 57 % and mean end-diastolic LV volume was 73 ml/m^2^. Out of the 203 patients, 39 (19 %) presented at least one asynergic myocardial segment on cine CMR. Hyper-T2 myocardial signal on fat suppressed black-blood spin echo T2-weighted CMR was present in 100 patients (62.5 %) with a mean extent of 10.9 ± 5.7 % of LV myocardial mass. Myocardial lesions on LGE were mostly limited in the posterolateral LV wall (60 %), often nodular (74 %), and located in the sub-epicardial layers (82 %) with a mean myocardial extent by planimetry of 11.4 ± 7.0 % of LV myocardial mass.

**Table 2:** Initial findings on CMR

LVEF at diagnosis, %	57.0 ± 9.0
LVEDV at diagnosis, ml/m^2^	72.9 ± 18.0
Patients with pericardial effusion (N,%)	58 (28.4)
Patients with asynergic myocardial segments (N,%)	39 (19)
LGE	
Number of patients with LGE (N,%)	203 (100)
Number of segments with LGE, N	3.8 ± 2.2
Myocardial extent of LGE (% myocardial surface area)	11.4 ± 7.0
Nodular Pattern (N,%)	150 (73.5)
Sub-epicardial lesions (N,%)	168 (82.3)
Midwall lesions (N,%)	33 (16.1)
Transmural lesions (N,%)	2 (1.0)
Presence of LGE in opposed walls (N,%)	77 (38.0)
Postero-lateral localization (N,%)	122 (60)
Anterior wall involved (N,%)	17 (8.3)
Postero-lateral and septal localisation (N,%)	60 (29.4)
Hyper T2 signal	
Number of patients with hyper T2 (N,%)	100 (62.5)
Number of segments with hyper T2 signal	1.9 ± 1.8
Myocardial extent of Hyper T2 (% myocardial surface area)	10.9 ± 5.7
Presence of T2 hyper-signal in opposed walls (N,%)	34 (21.2)
EGE	
Number of patients with EGE (N,%)	114 (56)
Number of segments with EGE	1.8 ± 1.9
Myocardial extent of EGE (% myocardial surface area)	8.6 ± 5.4
Presence of local EGE in opposed walls (N,%)	30 (14.7)

Values are expressed as mean ± SD unless specified

### Follow-up data

The clinical follow-up was assessed after a mean period of 18.9 ± 8.2 months. Out of the 203 patients, 22 experienced at least one major cardiovascular event (10.8 %) during follow-up. A total of 31 major cardiovascular events occurred during the study period and their characteristics are detailed in Table 3. LVEF at follow-up was greater than 50 % in 88.7 % of patients. Mean clinical score at follow-up was 0.6 ± 1.1, and 57 patients (28 %) had a clinical score >1 at follow-up.

**Table 3:** Mayor Adverse Clinical Events (MACE) at follow-up

Number of patients with MACE (N,%)	22 (10.8 %)
Hospitalization for cardiac causes	8
Heart failure	4
Ventricular Tachycardia	8
Recurrence of myocarditis	11
Cardiac transplantation or Death or Aborted sudden cardiac death	0

### CMR predictors of outcome

#### Primary endpoints

**Fig. 1:**
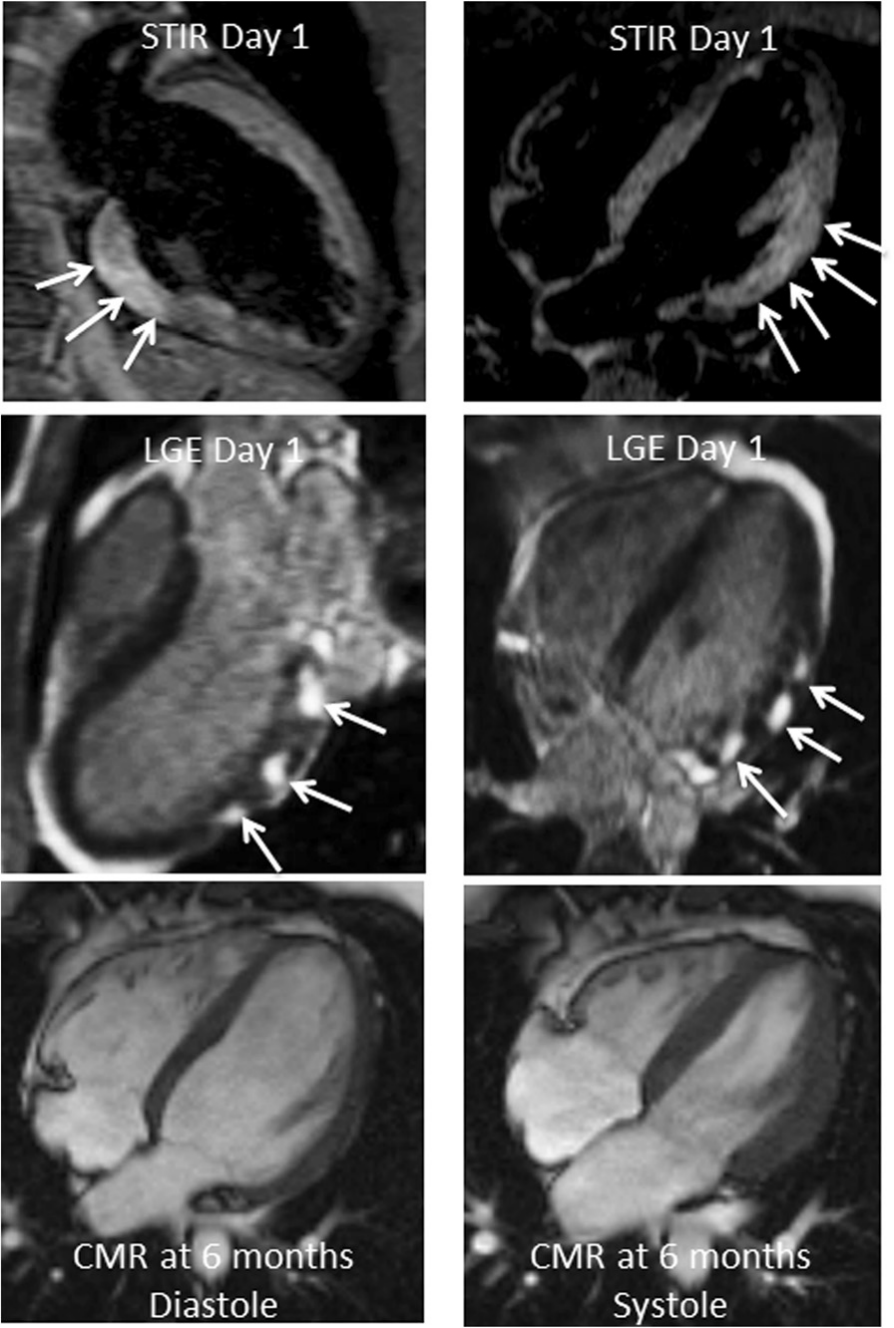
CMR data of a 25 year-old male performed at day 1 after the onset of an acute coronary-like syndrome (chest pain, ST segment alterations, mild troponin elevation). Black blood T2-weighted STIR CMR indicates the presence of segmental diffuse myocardial edema of the LV posterior and lateral walls representing 23 % of LV mass (top panel, arrows). Late gadolinium-enhanced CMR shows diffuse subepicardial nodular lesions in the posterolateral and lateral walls of the LV (11 % of LV mass) indicative of acute myocarditis (mid panel, arrows). Cine-MR at day 1 and 6 months (bottom panel) showed normal systolic global and segmental LV function (LV end-diastolic volume index 71 ml/m^2^, LV ejection fraction 61 %; 69 ml/m^2^ and 63 %, respectively). The patient did well and had no MACE during follow-up

**Fig. 2:**
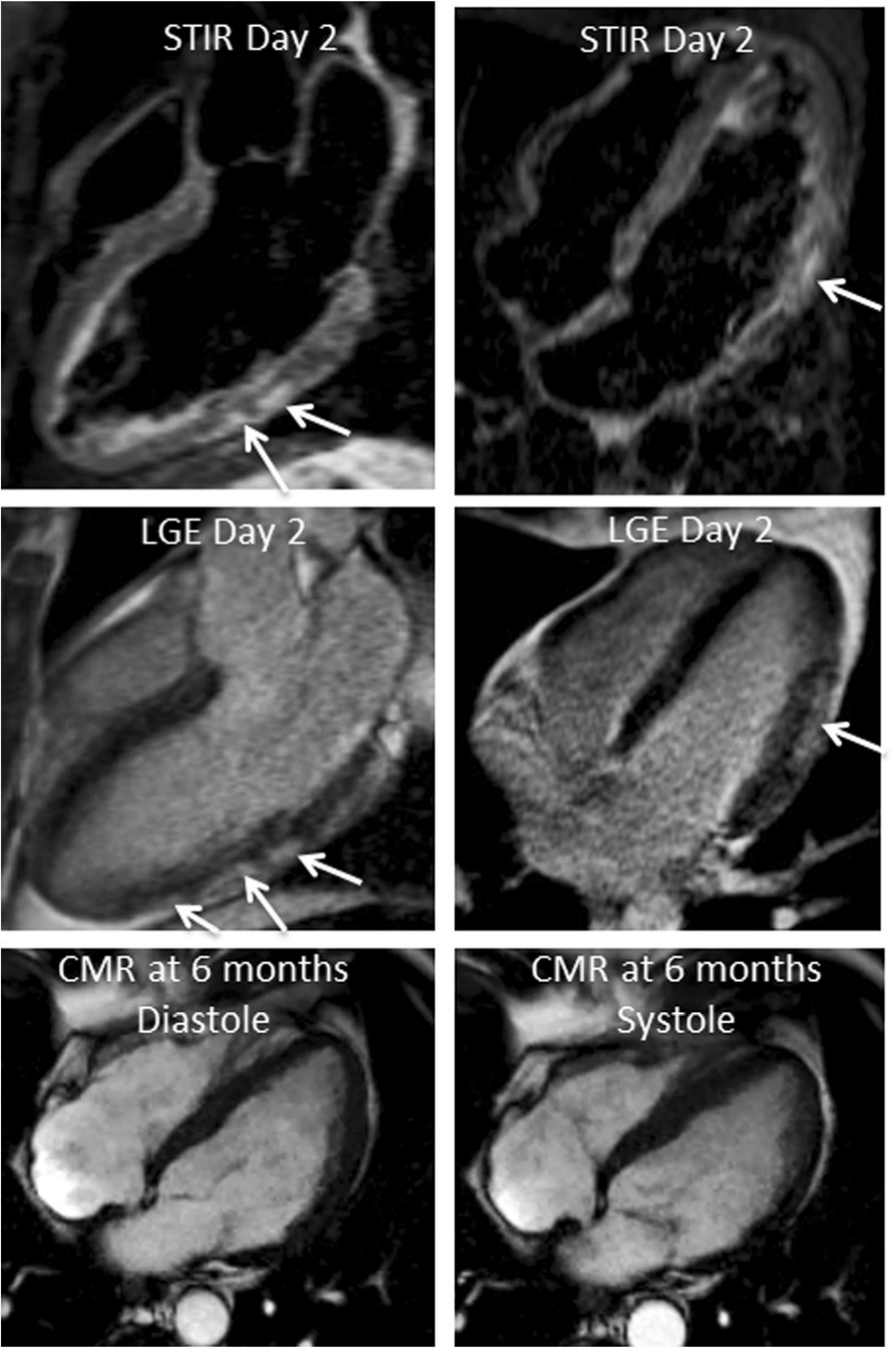
CMR data findings of a 56-year-old male performed at day 2 after the onset of an acute coronary-like syndrome (chest pain, ST segment alterations, mild troponin elevation). Black blood T2-weighted STIR CMR indicates the presence of limited subepicardial hypersignal in the mid portion of the posterolateral and lateral LV walls, indicative of small foci of myocardial edema (top panel, arrows, 4 % LV mass). Late gadolinium-enhanced CMR shows limited subepicardial nodular lesions in the mid portion of the posterolateral and lateral LV walls (mid panel, arrows, 6 % LV mass), indicative of acute myocarditis. Cine-MR at day 2 and 6 months (bottom panel) showed altered systolic global LV function (LV end-diastolic volume index 83 ml/m^2^, LV ejection fraction 45 %; 101 ml/m^2^ and 36 %, respectively). Despite optimal medical therapy, the patient suffered NYHA Class III heart failure during follow-up with diffuse LV hypokinesia predominant in the lateral wall

By univariate analysis, the presence and extent of early gadolinium enhancement were inversely related to the occurrence of MACE (mean number of myocardial segments exhibiting EGE 1.9 ± 1.9 in patients without MACE vs. 1.0 ± 1.7 in patients with MACE, *p* = 0.04; and mean myocardial extent of EGE 5.1 ± 6.0 % of LV myocardial mass vs. 2.2 ± 4.1 %, respectively, *p* = 0.0056) (Table 4). Although not significant, the extent of T2 lesions on initial black-blood T2-weighted CMR was also greater in patients without MACE relative to patients with subsequent MACE (7.0 ± 7.0 % vs. 4.9 ± 4.9 % of LV mass, respectively, *p* = 0.16) (Figs. 1 and 2). A lower initial LVEF was associated with adverse clinical outcome (57.6 ± 8.9 % in patient without MACE vs. 51.9 ± 9.0 % in patients with MACE, *p* = 0.009). No relationship was found between the extension, patterns (nodular/linear), localisation and wall distribution of LGE lesions and the occurrence of MACE (Figs. 1 and 2). By multivariate analysis, the only independent CMR predictor of adverse clinical outcome at follow-up was an initial alteration of LVEF (Table 5).

**Table 4:** Univariate analysis for CMR predictors of Major Adverse Cardiac Events

Variables	Presence of MACE at FU (*N* = 22)	Absence of MACE at FU (*N* = 181)	*P* value	OR (95 % CI)
CMR Pattern			*P* = 0.094	2.16 (0.86–5.38)
Nodular	13	137		
Linear	9	44		
Wall distribution			*P* = 0.103	
Subepicardial	15	153		
Midwall	7	26		
Transmural	0	2		
Localization			*P* = 0.65	
Posterolateral	13	109		
Septal	1	2		
Anterior	2	15		
Septal + posterolateral	6	54		
Presence of pericardial effusion	9	49	*P* = 0.18	1.86 (0.75–4.64)
Gender			*P* = 0.87	
Male	137	17		
Female	44	5		
Presence and extent of Early Gadolinium Enhancement (% myocardial surface area)	2.2 % ± 4.1	5.1 % ± 6.0	*P* = 0.005	
Number of segments with EGE	1.0 ± 1.7	1.9 ± 1.9	*P* = 0.04	
Age	47 ± 13	42 ± 17	*P* = 0.14	0.91 (0.32–2.62)
Presence of asynergic segments on CMR	6	33	*P* = 0.31	1.68 (0.61–4.62)
Presence of EGE in 2 opposed walls	3	27	*P* = 0.87	0.90 (0.25–3.25)
Presence of LGE in 2 opposed walls	8	69	*P* = 0.87	0.93 (0.37–2.32)
Myocardial extent of Late Gadolinium Enhancement (% myocardial surface area)	11.9 % ± 6.8	11.3 % ± 7.2	*P* = 0.72	
Number of segments with LGE	4.2 ± 2.4	3.7 ± 2.2	*P* = 0.41	
Presence of T2-hypersignal	8	92	*P* = 0.69	0.81 (0.27–2.46)
Presence of T2-hypersignal in opposed walls	2	32	*P* = 0.50	0.59 (0.13–2.79)
Myocardial extent of hyper T2 signal (% myocardial surface area)	4.9 ± 4.9	7.0 ± 7.0	*P* = 0.16	
Number of segments with hyper T2 signal	1.9 ± 1.8	1.9 ± 1.9	*P* = 0.9	
Initial end-diastolic LV volume, ml/m^2^	77 ± 22	73 ± 18	*P* = 0.39	
Initial LV ejection fraction, %	51.9 ± 9	57.6 ± 8.9	*P* = 0.009	

Data are presented as mean value, ± Standard Deviation

**Table 5:** Multivariate analysis for CMR predictors of Major Adverse Cardiac Events

Variables	Presence of MACE at FU (*N* = 22)	Absence of MACE at FU (*N* = 181)	*P* value	HR (95 % CI)
Presence and extent of Early Gadolinium Enhancement (% myocardial surface area)	2.2 % ± 4.1	5.1 % ± 6.0	*P* = 0.322	0.93 (0.80–1.07)
Age	47 ± 13	42 ± 17	*P* = 0.847	0.85 (0.96–1.03)
Myocardial extent of hyper T2 signal (% myocardial surface area)	4.9 % ± 4.9	7.0 % ± 7.0	*P* = 0.601	0.97 (0.86–1.08)
Initial LVEF, %	51.9 ± 9.0	57.6 ± 8.9	*P* = 0.04	0.89 (0.80–0.98)

#### Secondary endpoints

There was an inverse association between the extent of T2 lesions and the persistence or occurrence of clinical symptoms during follow-up (mean extent of T2 lesions 7.5 ± 7.4 % of LV mass in asymptomatic vs. 4.8 ± 5.1 % in symptomatic patients, *p* = 0.01). Also, an initial alteration of LVEF was associated to the presence of clinical symptoms at follow-up (55.0 ± 8.2 % in patients presenting symptoms at follow-up vs. 57.8 ± 9.3 % in asymptomatic, *p* = 0.04). Initial alteration of LVEF at diagnosis was found to be significantly associated to an abnormal EF at follow-up (patients with LVEF < 50 % at follow-up had an initial LVEF of 47.0 ± 10.7 %, vs. an initial LVEF of 57.3 ± 9.0 % in patients with follow-up LVEF ≥50 %; *p* = 0.001). A larger LV end-diastolic volume at initial presentation was associated to altered LVEF at follow-up (85.9 ± 21.7 ml/m^2^ in patients with altered follow-up LVEF vs. 71.8 ± 17.1 ml/m^2^ in those with preserved LVEF, *p* = 0.02). No relationship was found between the extension, patterns (nodular/linear), localisation and wall distribution of LGE lesions and subsequent alterations of LV functional parameters. Similarly, no association was found between the extent of EGE or T2 lesions and LVEF (*P* = NS).

## Discussion

In this prospective series of 203 consecutive patients presenting with a CMR-based diagnosis of AM, the initial CMR data including the presence and extent of acute focal myocardial edema and the extent of myocardial tissue damage on LGE-CMR were not independently related to clinical outcome. By multivariate analysis, the only independent predictor of outcome on CMR was the alteration of LV ejection fraction at the initial CMR performed early after the onset of AM.

The current data highlight the value of global LV systolic function as an independent predictor of outcome in AM and are in good agreement with that from Caforio et al. who reported that biventricular dysfunction at diagnosis was the main predictor of death/transplantation in patients with myocarditis [7]. Accordingly, Anzini et al. showed that baseline LV function was a marker for prognosis in a series of 82 patients with AM regardless of the clinical pattern of disease onset [9]. Because only a small number of patients underwent CMR in this study, potential CMR predictors could not be evaluated. The current study provides a prospective analysis of CMR predictors of long term outcome in a relatively large population of consecutive patients with suspected AM. To participate in the study, patients had to undergo CMR at the acute phase of AM, leading to the exclusion of the most severe, in particular those with hemodynamic compromise. However, this resulted in the exclusion of only 3 patients during the study period. Therefore, it should be emphasized that the main findings of the present study are valid in a population of patients with CMR-based diagnosis of AM in absence of severe heart failure, which is by far the most frequent presentation in clinical practice. In addition, it has been shown that fulminant myocarditis is a distinct clinical entity with an excellent long-term prognosis in patients who survive after the acute phase of AM [22]. Therefore, the determination of long term predictors of outcome in those patients may be less needed. The characteristics of our study population may account for the apparent conflicting results reported by Grün et al., where the authors studied a cohort of 203 patients with biopsy-proven myocarditis and found that the presence of LGE was an independent predictor of long-term all-cause mortality and cardiac mortality [21]. It is important to point out that the characteristics of the study population were quite different, since a significant proportion of patients presented with heart failure (almost 45 % of patients presenting with an advanced NYHA class III or IV, and more than 76 % presenting with NYHA II-III or IV Class) and altered LV functional parameters (mean LVEF of 45 %). It has been shown that viral genomes are frequently detected in EMBs of patients with systolic LV dysfunction and that myocardial persistence of various viruses, often presenting as multiple infections, may play a role in the transition from myocarditis to dilated cardiomyopathy [23], or as a precipitating factor for heart failure [15]. Also, patients of the current sudy were included on the basis of a LGE positive CMR and therefore the presence of LGE per se could not be evaluated as a potential predictor of outcome. As previously mentioned, the presence/extent of myocardial edema and EGE, as well as the extent of LGE and parameters of LV function, were assessed.

In the current study, the diagnosis of acute myocarditis was based on the acute clinical presentation (mostly acute chest pain) with electrocardiographic and/or biological data, and CMR that showed nodular or linear intra-myocardial lesions highly suggestive of acute nonischemic tissue damage. The criteria for the diagnosis of AM on CMR were slightly different from the established Lake-Louise criteria where AM is present when 2 out of 3 parameters (increased signal on T2, early enhancement and late-enhancement) are met [13]. In our study, lesions of LGE had to be present with typical patterns of AM because this parameter is more specific and could limit the inclusion of patients with myocardial edema of other causes. For the same reason, we did not include patients with clinically suspected acute myocarditis but negative CMR because we believe that the diagnosis often remains uncertain in those cases and this might have led to an inhomogeneous group of patients. The diagnosis of AM was not proven by biopsy in agreement with practice Guidelines, since only 5 % of the patients presented with mild to moderate heart failure and responded to conventional medical therapy [10]. Patients with hemodynamic compromise were not included and the study confirms that acute myocarditis has an excellent prognosis in routine consecutive patients [1, 2]. It should also be emphasized that the presence of a typical pattern of LGE on initial CMR was an inclusion criterion, therefore the presence/absence of LGE could not be evaluated as a potential predictor of outcome.

Although not significant, there was a clear trend between the presence and extent of myocardial edema on initial T2-weighted CMR and a lower rate of subsequent MACE, as well as less persistent clinical symptoms during follow-up. Recently, McLellan et al. reported concordant results in patients with acute onset cardiomyopathy, showing that the amount of myocardial inflammation identified by an elevated global relative enhancement (GRE) could predict recovery of LV function [20]. However, the acquisition sequences for depiction of myocardial edema were different in those 2 single-centre studies, and other studies will be needed to confirm or infirm this finding. The lesions of EGE were not assessed by the EGE ratio (EGEr) with normalization to the signal of the skeletal muscle, but with the use of a threshold of 4 SD above the mean signal of remote myocardium [13]. In the 2009 JACC white paper, it is stated that the EGE may be visually appreciated and that there is no demonstration of the diagnostic superiority of EGEr over the visual assessment for the diagnosis of AM [13]. Also, the patients enrolled in the study were non severe patients and the probability to have diffuse and homogeneous inflammation of the myocardium was weak.

Overall, the current study confirms and highlights the favorable outcome of patients presenting with initially non severe AM and no hemodynamic compromise. For this reason, the combined clinical primary end point was broad and no patient experienced a hard event (transplantation, cardiac death or aborted cardiac death). Recently, Schumm et al. reported that patients presenting with suspected clinical myocarditis and normal CMR scans had favorable outcomes [24].

In our sudy population, the lower extent of EGE as compared to LGE might appear to be in contradiction to the sole physiology of gadolinium kinetics within the myocardium. As stated in the White JACC paper, T2 sequences for depiction of regional edema may have a limited sensitivity in less severe inflammation [13]. It is likely that in the studied homogeneous population of patients presenting with unsevere acute myocarditis and no hemodynamic compromise (by far the most frequent patients presenting with AM), the lack of sensitivity of conventional sequences (either T2-STIR or SSFP cines) in such unsevere AM, as the lack of contrast at least for SSFP, may account for the lower extent of lesions in such sequences as compared to LGE, which by basics principles is set to optimize contrast.

Finally, an ischemic etiology was ruled out in a significant proportion of patients based upon clinical parameter assumption and CMR characteristics. This is a limitation of the study since no specific examination was performed to formally rule out coronary disease. The study did not provide comparisons between biomarkers and myocardial lesions on CMR in relation to the clinical outcome. Also, the empirical medical regimen based on betablockers and ACE inhibitors might have had some influence on patient outcomes and this impact is largely unknown.

## Conclusions

In routine consecutive patients without severe hemodynamic compromise and a CMR-based diagnosis of acute myocarditis, various CMR parameters such as the presence and extent of myocardial edema and the extent of late gadolinium-enhanced LV myocardial lesions were not predictive of outcome. The only independent CMR predictor of adverse clinical outcome at follow-up was an initial alteration of LVEF.

## Abbreviations

CMR: Cardiovascular Magnetic Resonance
MACE: Major Adverse Clinical Events
ECG: Electrocardiogram
LV: Left ventricular
LVEF: Left ventricular ejection fraction
AM: Acute myocarditis
EMB: Endomyocardial biopsy
SSFP: Steady State Free Precession
EGE: Early gadolinium enhancement
EGEr: Early gadolinium enhancement ratio
GRE: Global Relative Enhancement
LGE: Late gadolinium enhancement
TI: Inversion time
MI: Myocardial infarction
